
JOURNAL INFORMATION
==============================
NLM Title Abbreviation: ScientificWorldJournal
Journal ID: ScientificWorldJournal
NLM Journal ID: 101131163
Journal ID: TSWJ

The Scientific World Journal

ISSN: 2356-6140
EISSN: 1537-744X
Publisher: Wiley

ARTICLE INFORMATION
==============================
PMCID: PMC6084002
PMID: 30147619
DOI: 10.1100/tsw.2001.233
Article ID: 485796
Article version: 1
Subjects: Short Report

The Late Increase of Free Radicals During Genotoxic-Stress Induced Apoptosis is Associated with Cytochrome C Release From Mitochondria Induced by Caspase-Mediated Feedback Loop Amplification

Chen Quan 1 2
Chai Yucheng 3
Mazuder Suparna 1
Drazba Judy 5
Chisolm Guy 3
Almasan Alex 1 4
1 Department of Cancer Biology, The National Key Laboratory of Biomembrane and Membrane Biotechnology, Chinese Academy of Sciences, Beijing 100080, China
2 The Cleveland Clinic Foundation, Cleveland, OH 44195, USA
3 Department of Cell Biology, The National Key Laboratory of Biomembrane and Membrane Biotechnology, Chinese Academy of Sciences, Beijing 100080, China
4 Lerner Research Institute, and Department of Radiation Oncology, The National Key Laboratory of Biomembrane and Membrane Biotechnology, Chinese Academy of Sciences, Beijing 100080, China
5 Department of Imaging Facility, The National Key Laboratory of Biomembrane and Membrane Biotechnology, Chinese Academy of Sciences, Beijing 100080, China
Publication date: 2001
Electronic publication date: 2001 Nov 2
Volume: 1
First page: 142
Last page: 142
Copyright: Copyright © 2001 Quan Chen et al.
Copyright year: 2001
License: This is an open access article distributed under the Creative Commons Attribution License, which permits unrestricted use, distribution, and reproduction in any medium, provided the original work is properly cited.
License URL: https://creativecommons.org/licenses/by/3.0/

==============================
Miami *Nature Biotechnology* Short Reports
*TheScientificWorld* (2001) 1 (S3), 142SR
ISSN 1532-2246; DOI 10.1100/tsw.2001.233

### THE LATE INCREASE OF FREE RADICALS DURING GENOTOXIC-STRESS INDUCED APOPTOSIS IS ASSOCIATED WITH CYTOCHROME C RELEASE FROM MITOCHONDRIA INDUCED BY CASPASE-MEDIATED FEEDBACK LOOP AMPLIFICATION

Quan Chen (1,2), Yucheng Chai (3), Suparna Mazuder (1), Judy Drazba (5), Guy Chisolm (3), and Alex Almasan (1,4,#)

Department of Cancer Biology (1), Department of Cell Biology (3), Lerner Research Institute, and Department of Radiation Oncology (4), Department of Imaging Facility (5), The Cleveland Clinic Foundation, Cleveland, OH 44195; (2)The National Key Laboratory of Biomembrane and Membrane Biotechnology, Chinese Academy of Sciences, Beijing 100080, China

Release of cytochrome c (cyt c) from mitochondria is a critical event for the onset of apoptosis induced by death stimuli. We have described recently that cyt c was released in two distinct stages and that a positive feedback loop linked caspase activation to the late cyt c release and mitochondrial dysfunction during genotoxic stress-induced apoptosis (*Cell Death and Differentiation* (1999) 7(2), 227-233). We show here that there are also distinct levels of intracellular reactive oxygen species (ROS), namely hydrogen peroxide and superoxide anion, measured by CM-H2DCFDA and dehydroethidium staining as determined by flow cytometry following ionizing radiation (IR) in IM-9 cells. There was a small increase of ROS and glutathione consumption in both parental IM-9 and its derivative Bcl-2 over-expressing cells following IR. The late cellular ROS increase was associated with the cyt c depletion from mitochondria by caspase-mediated feedback amplification. We showed that the late ROS production and GSH depletion were inhibited by z-VAD-fmk and ectopic Bcl-2. U266, another multiple myeloma cell line which is resistant to radiation-induced apoptosis shows no increase of ROS following IR. Furthermore, over-expression of a dominant negative caspase-9 prevented IR-induced cell death, the increase of cellular ROS levels, and GSH depletion. While ectopic Bcl-2 prevents the late ROS production and depletion of GSH, endogenous Bcl-2 could be targeted by caspases and its cleavage is associated with the late stage cyt c release and increase of cellular ROS, since over-expression of truncated Bcl-2 induces cyt c release, and consequently apoptosis. These results suggest that intracellular ROS production is associated with the late cyt c release and that Bcl-2 truncation is a component of the amplification loop of caspase-mediated cyt c release and cellular ROS production.
